# Autophagy is induced in the skeletal muscle of cachectic cancer patients

**DOI:** 10.1038/srep30340

**Published:** 2016-07-27

**Authors:** Zaira Aversa, Fabrizio Pin, Simone Lucia, Fabio Penna, Roberto Verzaro, Maurizio Fazi, Giuseppina Colasante, Andrea Tirone, Filippo Rossi Fanelli, Cesarina Ramaccini, Paola Costelli, Maurizio Muscaritoli

**Affiliations:** 1 Department of Clinical Medicine, Sapienza University of Rome, Rome, Italy; 2 Department of Clinical and Biological Sciences, University of Turin, Turin, Italy; 3 Interuniversity Institute of Myology, Italy; 4 Department of Surgery, M.G. Vannini Hospital, Rome, Italy; 5 UOSA Chirurgia Bariatrica, Azienda Ospedaliera Universitaria Senese, Siena, Italy

## Abstract

Basal rates of autophagy can be markedly accelerated by environmental stresses. Recently, autophagy has been involved in cancer-induced muscle wasting. Aim of this study has been to evaluate if autophagy is induced in the skeletal muscle of cancer patients. The expression (mRNA and protein) of autophagic markers has been evaluated in intraoperative muscle biopsies. Beclin-1 protein levels were increased in cachectic cancer patients, suggesting autophagy induction. LC3B-I protein levels were not significantly modified. LC3B-II protein levels were significantly increased in cachectic cancer patients suggesting either increased autophagosome formation or reduced autophagosome turnover. Conversely, p62 protein levels were increased in cachectic and non-cachectic cancer patients, suggesting impaired autophagosome clearance. As for mitophagy, both Bnip3 and Nix/Bnip3L show a trend to increase in cachectic patients. In the same patients, Parkin levels significantly increased, while PINK1 was unchanged. At gene level, Beclin-1, p-62, BNIP3, NIX/BNIP3L and TFEB mRNAs were not significantly modulated, while LC3B and PINK1 mRNA levels were increased and decreased, respectively, in cachectic cancer patients. Autophagy is induced in the skeletal muscle of cachectic cancer patients, although autophagosome clearance appears to be impaired. Further studies should evaluate whether modulation of autophagy could represent a relevant therapeutic strategy in cancer cachexia.

Cancer patients frequently experience cachexia, a complex multifactorial syndrome associated to serious clinical consequences^1^. Muscle wasting is a major feature in cancer cachexia and negatively affects patients’ outcome, quality of life and response to anti-neoplastic treatments^2^,^3^. The mechanisms underlying muscle wasting in cancer cachexia are still not completely understood, although several studies suggest that hyperactivation of cellular degradative pathways, such as the ubiquitin proteasome system, plays a major role^2^. Recently also the autophagic degradation has been suggested to be involved in the pathogenesis of muscle wasting under different catabolic conditions including cancer^4^,^5^. Macroautophagy (hereafter referred to as autophagy) is a highly conserved homeostatic mechanism involved in the lysosomal-dependent degradation of cellular constituents including bulk cytoplasm, long-lived or misfolded proteins, damaged organelles, toxic protein aggregates and intracellular pathogens. Autophagy constantly works at basal levels in all eukaryotic cells to ensure a quality-control of cytoplasmic components and prevent accumulation of degenerated protein and organelles^6^,^7^. However, under marked nutrient restriction and other stress conditions, autophagy is rapidly up-regulated in order to replace old or damaged cellular constituents, recycle biomolecules for the synthesis of new components and mobilize cellular energy stores^8^,^9^.

In the initial step of autophagy, a small portion of cytoplasm, including organelles or soluble materials, is sequestered by an isolation membrane (phagophore) to form an autophagosome. The autophagosome then fuses with the lysosome to become an autolysosome and degrade the cargo contained within it^6^,^7^.

Autophagy was initially considered a non-selective degradation pathway of bulk cytoplasm, but increasing evidences have shown that it can be involved also in the selective removal of protein aggregates or specific organelles such as mitochondria via mithophagy, ribosomes via ribophagy, peroxisomes via pexophagy and many others^4^,^9^. The selectivity of autophagic degradation is conferred by specific signals such as p62, Bnip3, Nbr1, which have both a cargo-binding domain (that recognizes and attaches organelles) and a LC3-interacting region (LIR), that recruits and binds essential autophagosome membrane proteins^10^. Adaptor proteins are able to recognize their target by specific flag molecules or post-translational modifications, such as ubiquitylation, phosphorylation and acetylation, presented on the surface of the cargo^11^,^12^.

Recent findings suggest that autophagy plays a central role in the regulation of muscle homeostasis both under constitutive conditions and in response to various stimuli such as cellular stress, fasting or exercise^4^. Indeed, skeletal muscle is a crucial metabolic center, and an efficient autophagic flux is fundamental to guarantee a rapid and proper turnover of cell components^12^. The pivotal role played by autophagy in the regulation of skeletal muscle mass is underscored by the phenotypes of mice with muscle specific ablation of genes encoding autophagy-related proteins^13^. Indeed, muscle-specific deletion of a gene crucial for autophagy such as Atg7 resulted in profound muscle atrophy and age-dependent decrease in force^14^.

Alterations in autophagic degradation with accumulation of unfolded and aggregate-prone proteins and dysfunctional organelles is a typical feature of several myopathies^4^,^13^,^15^,^16^. Disorders in which autophagic vacuoles are seen in the skeletal muscle are generally referred to as authophagic vacuolar myopathies which include Pompe disease and Danon disease^17^. Recently, however, defective autophagy has been demonstrated to contribute also to the pathogenesis of different forms of muscular dystrophies which could display either accumulation of altered organelles inside myofibers (impaired autophagy), or excessive degradation of myofiber components (excess autophagy)^4^.

A modulation of autophagy has been reported in the skeletal muscle also during several conditions such as fasting^18^,^19^, exercise^20^, ageing^21^,^22^, sepsis^23^, denervation^24^, disuse^25^, critical illness^26^,^27^, cirrhosis^28^, COPD^29^,^30^ and cancer^21^. In this regard, autophagy has been shown to contribute to muscle atrophy in three different experimental models of cancer cachexia^21^ and a modulation of representative markers of this degradative pathway has been documented in the skeletal muscle of esophageal^31^, lung^32^, upper gastrointestinal and pancreatic^33^ cancer patients. At present, however, the role of autophagy in the pathophysiology of human cancer-related muscle loss is not yet fully elucidated. This is due, at least in part, to the impossibility to dynamically assess, in the clinical setting, the autophagic flux with the techniques currently available^34^.

Aim of the present study was to provide a comprehensive panel of autophagy and mitophagy markers, at both the gene and protein level, in order to better clarify to what extent autophagy is involved in the pathogenesis of cancer-induced muscle loss.

## Materials and Methods

### Patients

The methods were carried out in accordance with the approved guidelines and regulations. After approval of the local ethical committees at M.G. Vannini Hospital in Rome (Italy) and Azienda Ospedaliera Universitaria Senese in Siena (Italy), and after the obtainment of written informed consent, twenty-nine consecutive cancer patients (17 males and 12 females) were enrolled among those undergoing abdominal surgery (Table 1). Age and sex matched controls patients were recruited among those undergoing abdominal surgery for non-neoplastic diseases (6 males and 5 females). Reasons for abdominal surgery in controls were: incisional hernia, cholelithiasis, benign prostatic hyperplasia, epigastric hernia and mesenteric cyst.

Exclusion criteria for both cancer patients and controls were: liver failure, diabetes, metabolic acidosis, acute and chronic renal failure, sepsis, AIDS, inflammatory bowel diseases, acute and chronic hepatitis, autoimmune disorders and chronic obstructive pulmonary disease.

### Nutritional assessment and body composition evaluation

The nutritional assessment included anthropometric [height, actual body weight, % body weight loss (BWL) during the previous 6 months, body mass index (BMI), usual body weight], immunological (total lymphocyte count), and biochemical (serum albumin) indices. Blood samples were collected before the surgical procedure and analyzed in the hospital clinical laboratory to evaluate, besides total lymphocyte count and serum albumin, the following parameters: haemoglobin, white cell count, serum total protein, C-reactive protein, ferritin, serum iron, creatinine, total cholesterol, HDL cholesterol, LDL cholesterol and triglycerides.

The presence of anorexia was evaluated using the anorexia questionnaire^35^, that investigates the presence of early satiety, taste/smell alterations, meat aversion, nausea/vomiting. Patients reporting at least one of these symptoms were considered as anorectic. Patients were also asked to report on the visual analogic scale (VAS) their self-assessment of appetite. The VAS consisted of a line of 100 mm, the extremities being anchored to “hunger” (0 mm) and “no hunger” (100 mm).

Body composition was assessed by bioelectrical impedance analysis (BIA) in all patients included in the study.

Finally, patients were assessed for the presence of pre-cachexia or cachexia according to the current available diagnostic criteria^1^,^36^. In particular, pre-cachexia was defined according to the ESPEN Special Interest Group (SIG) on cachexia-anorexia in chronic wasting diseases consensus definition^36^ by the presence of all the following criteria:

- Weight loss ≤5% of usual body weight in the last 6 months;
- Systemic inflammatory response indicated by C-reactive protein (CRP) above the upper limit of normality for the method used (>0.5 mg/dl in this study);
- Anorexia or anorexia-related symptoms.

Patients were classified as cachectic when they showed BWL > 5% in the previous 6 months according the International Consensus definition on cancer cachexia^1^.

### Muscle biopsy

Biopsy specimens were obtained during the initial phase of the operation from the rectus abdominis muscle. After skin incision and dissection through the subcutaneous fat, the anterior sheet of the rectus abdominis muscle was opened with scissors and a muscle biopsy specimen was obtained (approximately 0.5 g). Small bleeding vessels were carefully controlled with ligatures and cautery after the muscle biopsy had been obtained; thereafter the operation continued in a routine fashion. No complications occurred from the biopsy procedure. Biopsy specimens were immediately frozen in liquid nitrogen and stored at −80 °C until analysis; part of the specimens was used for the present study and part was kept stored for further, subsequent investigations.

### Real-time PCR

Total RNA was obtained using TriReagent (Sigma Aldrich, St. Louis MO) following the manufacturer’s instructions. RNA concentration was determined fluorometrically using the RiboGreen reagent (Invitrogen, Carlsbad CA).

Total mRNA was retrotranscribed using the iScript cDNA synthesis kit (Bio-Rad, Hercules, CA). Transcript levels were determined by real-time PCR using the SsoFast EvaGreen Supermix and the MiniOpticon Thermal Cycler (Bio-Rad). Ten seconds of denaturation at 95 °C was followed by 30 seconds of annealing/extension at 60 °C and repeated for 40 cycles. Every qPCR was validated by analyzing the respective melting curve. Only one peak was detectable, indicating the presence of just one amplicon.

Gene expression was normalized to both GAPDH and TATA-binding protein (TBP) expression and calculated using the 2^−ΔΔCt^ method.

Primers sequences used (forward and reverse) are indicated in Supplementary Table S1 (see supplementary information).

### Western blotting

Approximately 50 mg of rectus abdominis muscle was homogenized in a buffer containing 10 mM HEPES pH 7.5, 10 mM MgCl_2_, 5 mM KCl, 0.1 mM EDTA pH 8.0, 0.1% TritonX-100, 1 mM dithiothreitol, 0.1 mM PMSF, with freshly added protease and phosphatase inhibitor cocktails; centrifuged at 3000 × *g* for 4 minutes at 4 °C; and the supernatant collected (cytosolic proteins). The pellet obtained was resuspended in a buffer containing 20 mM HEPES pH 7.9, 25% glycerol, 500 mM NaCl, 1.5 mM MgCl2, 0.2 mM EDTA pH 8.0, 0.5 mM dithiothreitol, 0.2 mM PMSF and freshly added protease and phosphatase inhibitor cocktails; kept on ice for 30 minutes (vortexing samples every 10 minutes); centrifuged at 3000 × *g* for 4 minutes at 4 °C; and the supernatant collected (nuclear proteins). Protein concentration was determined by using the Bradford reagent (Bio-Rad) with bovine serum albumin as standard.

Equal amount of proteins (30 μg) were heat denatured in sample loading buffer (50 mm Tris–HCl, pH 6.8, 100 mM DTT, 2% SDS, 0.1% bromophenol blue, 10% glycerol) resolved by SDS-page and transferred to Nitrocellulose or PVDF membrane (Bio-Rad). Protein transfer was checked by Ponceau-S staining. The filters were blocked with Tris-Buffered saline containing 0.05% Tween-20 and 5% non-fat dry milk and then were incubated overnight with the following primary antibodies: a rabbit polyclonal anti- human beclin-1 (B6186, Sigma, St. Louis, MO, USA), a rabbit polyclonal anti-human LC3B (#L7543, Sigma Aldrich), a rabbit polyclonal anti-human p62/SQSTM1 (#P0067, Sigma Aldrich), a rabbit polyclonal anti- human Bnip3 (#38621; Abcam, Cambridge, UK), a rabbit polyclonal anti-human Nix/Bnip3L (#N0399, Sigma Aldrich), a mouse monoclonal anti-human Parkin (#P6248, Sigma Aldrich), a goat polyclonal anti-human PINK1 (#SAB2500794, Sigma Aldrich) and a goat polyclonal anti-human TFEB (#2636, Abcam). A goat polyclonal anti-human GAPDH antibody (sc-20357, Santa Cruz Biotechnology, Santa Cruz, CA) and a rabbit polyclonal anti Lamin A antibody (sc-20680, Santa Cruz Biotechnology, Santa Cruz, CA) were used as loading control for cytosolic and nuclear extracts, respectively. Peroxidase conjugated IgG were used as secondary antibodies. Immunoreactive protein bands were detected by enhanced chemoluminescence on a photon sensitive film. Molecular weights of protein bands were determined by Dual Precision molecular weight standards (Bio-Rad). Quantification of the bands was performed by densitometric analysis using the software TotalLab (NonLinear Dynamics, New Castle on Tyne, UK). Since the western blotting were performed in the presence of DTT, only Bnip3 monomeric form could be detected^37^. Purity of the obtained nuclear and cytosolic fractions were checked by probing the membrane for Lamin A (nuclear) and tubulin (cytosolic; #T5168, Sigma Aldrich). A representative pattern is reported in Supplementary Figure S1. Although fractions were not totally pure, nuclear protein enrichment was considered acceptable. Indeed, Lamin A/tubulin ratio was markedly and significantly higher in the nuclear than in the cytosolic fractions.

### Statistics

Data are expressed as means ± standard error (SEM). Statistical analysis was performed by using Mann-Whitney or Kruskal Wallis test followed by post hoc test as appropriate. p < 0.05 was considered statistically significant.

## Results

Clinical characteristics of the subjects studied are shown on Table 1. The mean age of cancer and control patients was over 60 years (cancer patients, 68 ± 2 years; control patients 63 ± 4 years, p = n.s.), with a similar proportion of male and female subjects in the two groups. The mean BMI was not different between cancer and control patients and lied in the overweight zone for both groups. Despite this, cancer patients presented in the last six months an average percent of body weight loss of 6.7 ± 1.5 (range: 0–33%), and 38% of them complained anorexia-related symptoms.

When cancer patients were classified upon the concepts of the SIG-ESPEN and the International Consensus definition on cancer cachexia^1^,^36^, pre-cachexia was present in 3 cancer patients, cachexia in 12 cancer patients, while 14 cancer patients did not match either criteria and were considered non-cachectic. Since the small number of patients with pre-cachexia might have affected statistical analysis, we decided hereafter to merge pre-cachectic with non-cachectic cancer patients into one single group (n = 17).

Body composition assessed with BIA, showed that both fat free mass (FFM) and fat free mass index (FFMI) were significantly reduced in cachectic versus non-cachectic cancer patients, suggesting that anthropometric measurements correctly identified patients’ categories (Table 2).

All cancer patients (cachectic and non-cachectic) had mild anemia, with haemoglobin levels significantly reduced with respect to controls (Table 3). Interestingly and not surprisingly, only cachectic cancer patients had significantly lower serum albumin and total lymphocytes count when compared to controls (Table 3), while C-reactive protein levels, were increased in both non-cachectic and cachectic cancer patients, although this difference did not reach statistical significance (Table 3). All the other biochemical parameters evaluated were not statistically different among the study groups (Table 3).

To estimate the activation of the autophagic-lysosomal degradative pathway, we assessed muscle expression of proteins commonly recognized as autophagy markers^34^ in rectus abdominis muscle biopsies obtained intraoperatively from surgical cancer and control patients.

**Figure 1:**
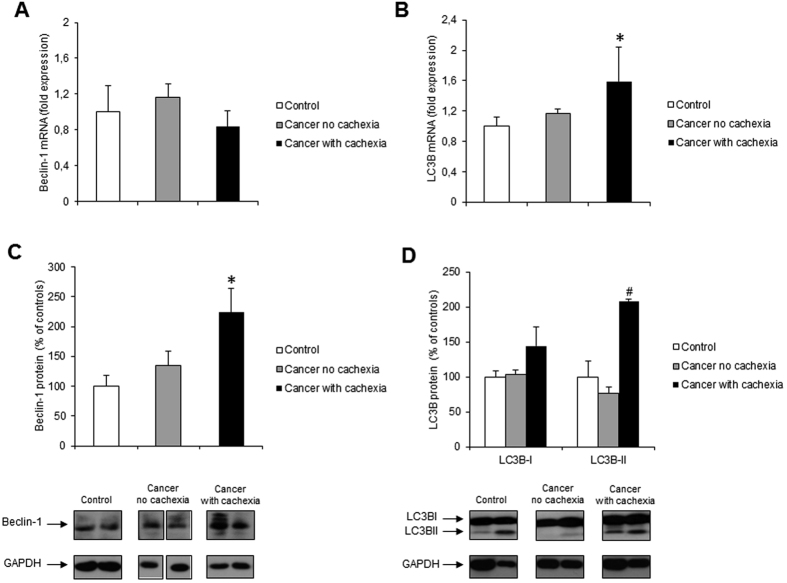
Beclin-1 and LC3B expression in rectus abdominis muscle of cancer and control patients. (**A**) Beclin-1 and (**B**) LC3B mRNA levels were evaluated by real-time PCR (control, n = 11; non cachectic cancer patients, n = 17; cachectic cancer patients, n = 12); (**C**) Beclin-1 and (**D**) LC3BI and LC3BII protein levels were evaluated by western blotting (control, n = 9; non cachectic cancer patients, n = 16; cachectic cancer patients, n = 10): representative western blots for Beclin-1, LC3BI, LC3BII and GAPDH (loading control) are shown on the lower panel and densitometric quantifications of Beclin-1, LC3B-I and LC3B-II protein levels normalized to GAPDH are shown on the upper panel. Representative pattern for Beclin-1, group ‘Cancer no cachexia’ consists of two non adjacent lanes on the same blot. The whole blot is reported in Supplementary Figure S2. Data (mean ± SEM) are expressed as percentage of controls. Significance of the differences: *p < 0.05 vs controls; ^#^p < 0.05 vs cancer no cachexia.

We first evaluated the expression of Beclin-1, a protein essential for the initiation of autophagy, the levels of which are considered a marker of autophagy induction^38^,^39^. Beclin-1 mRNA levels were unchanged (Fig. 1A), while Beclin-1 protein expression was significantly increased in the skeletal muscle of cachectic cancer patients (Fig. 1C) suggesting autophagy activation.

We next evaluated the expression of the microtubule-associated protein 1 light chain 3B (LC3B), a ubiquitin-like molecule that is the mammalian homologue of the yeast Atg8 and is essential for autophagosome formation^40^. LC3B mRNA levels were significantly increased in cachectic cancer patients (Fig. 1B) suggesting transcriptional induction. As LC3B before targeting the autophagosome undergoes post-translational modifications that influence its activity and are used as marker of autophagosome formation^34^, we next evaluated protein expression of the LC3B isoform I (LC3BI), that resides in the cytosol and has a carboxyterminal glycine, and the lipidated isoform II (LC3B-II), that is tightly associated to autophagosome membrane and results by conjugation of LC3B-I with phosphatidylethanolamine^41^. We found that LC3B-I protein levels were not significantly modified in cancer patients, while LC3B-II protein levels were significantly increased in cachectic cancer patients (Fig. 1D) suggesting either autophagosome formation or reduced autophagosome turnover. Unfortunately, it was not possible to discern this point by means of flux experiments using autophagy inhibitors such as colchicine or bafilomycin A1^42^,^43^, being this a study on human muscle biopsies.

**Figure 2:**
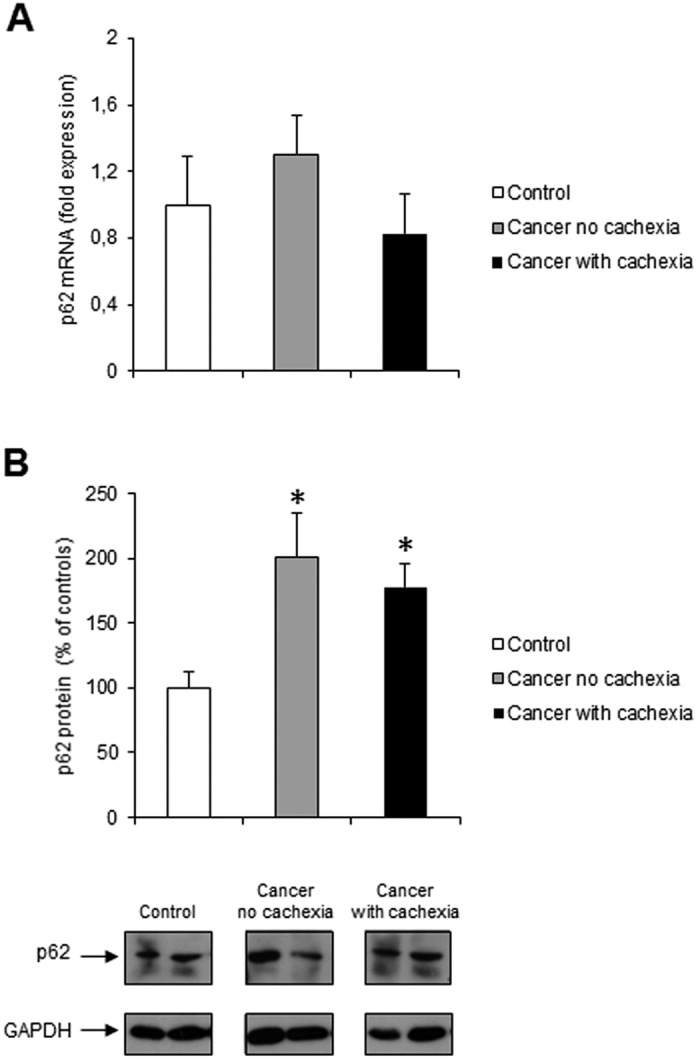
p62 expression in rectus abdominis muscle of cancer and control patients. (**A**) p62 mRNA levels were evaluated by real-time PCR (control, n = 11; non cachectic cancer patients, n = 17; cachectic cancer patients, n = 12); (**B**) p62 protein levels were evaluated by western blotting (control, n = 8; non cachectic cancer patients, n = 15; cachectic cancer patients, n = 10): representative western blots for p62 and GAPDH (loading control) are shown on the lower panel and densitometric quantifications of p62 protein levels normalized to GAPDH are shown on the upper panel. Data (mean ± SEM) are expressed as percentage of controls. Significance of the differences: *p < 0.05 vs controls.

Thus, we proceeded with the evaluation of p62/SQSTM1 (hereafter referred to as p62), an adaptor molecule involved in selectively targeting protein aggregates to autophagosomes by simultaneously binding LC3B and ubiquitinated proteins^44^. Since p62 is constantly removed by autophagy, it is considered a good marker of autophagic vescicle turnover^34^. p62 mRNA levels were not significantly modified (Fig. 2A), while p62 protein levels were increased in all cancer patients (Fig. 2B) suggesting impaired autophagosome clearance, possibly due to exhaustion of the lysosomal degradative capacity.

**Figure 3:**
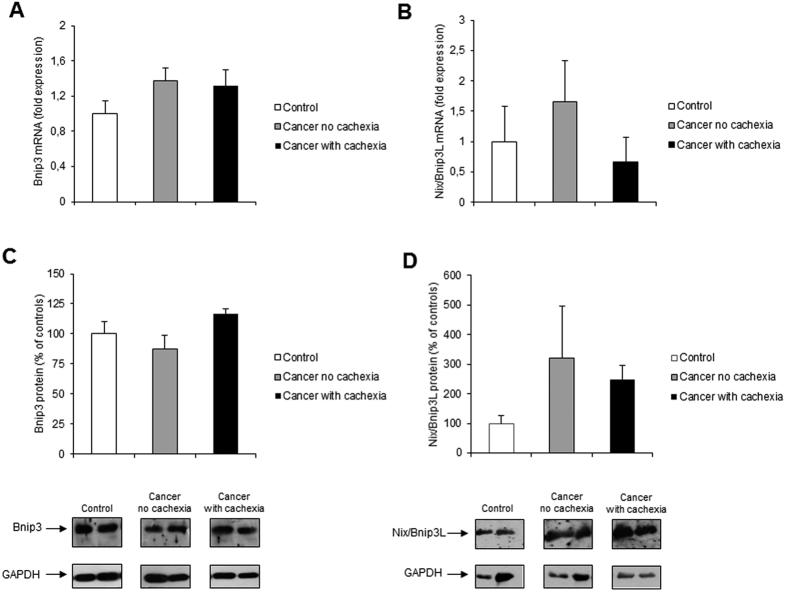
Bnip3 and Nix/Bnip3L expression in rectus abdominis muscle of cancer and control patients. (**A**) Bnip3 and (**B**) Nix/Bnip3L mRNA levels were evaluated by real-time PCR (Bnip3: control, n = 8; non cachectic cancer patients, n = 16; cachectic cancer patients, n = 10; Nix/Bnip3L: control, n = 9; non cachectic cancer patients, n = 16; cachectic cancer patients, n = 11); (**C**) Bnip3 and (**D**) Nix/Bnip3L protein levels were evaluated by western blotting (Bnip3: control, n = 11; non cachectic cancer patients, n = 17; cachectic cancer patients, n = 10; Nix/Bnip3L: control, n = 3; non cachectic cancer patients, n = 5; cachectic cancer patients, n = 5): representative western blots for Bnip3, Nix/Bnip3L and GAPDH (loading control) are shown on the lower panel and densitometric quantifications of Bnip3 and Nix/Bnip3L protein levels normalized to GAPDH are shown on the upper panel. Data (mean ± SEM) are expressed as percentage of controls.

**Figure 4:**
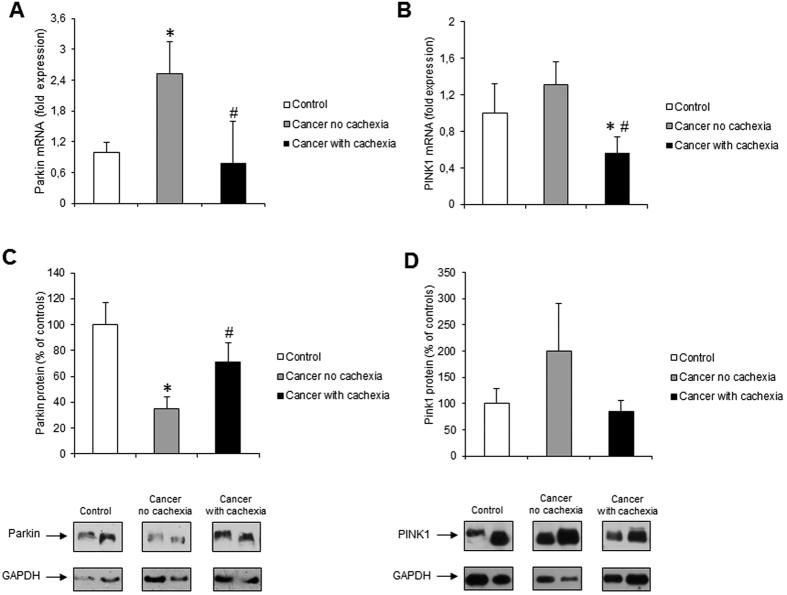
PINK1 and Parkin expression in rectus abdominis muscle of cancer and control patients. (**A**) PINK1 and (**B**) Parkin mRNA levels were evaluated by real-time PCR (PINK1: control, n = 9; non cachectic cancer patients, n = 16; cachectic cancer patients, n = 11; Parkin: control, n = 9; non cachectic cancer patients, n = 16; cachectic cancer patients, n = 11); (**C**) PINK1 and (**D**) Parkin protein levels were evaluated by western blotting (PINK1: control, n = 7; non cachectic cancer patients, n = 8; cachectic cancer patients, n = 9; Parkin: control, n = 7; non cachectic cancer patients, n = 8; cachectic cancer patients, n = 10): representative western blots for PINK1, Parkin and GAPDH (loading control) are shown on the lower panel and densitometric quantifications of PINK1 and Parkin protein levels normalized to GAPDH are shown on the upper panel. Data (mean ± SEM) are expressed as percentage of controls. Significance of the differences: *p < 0.05 vs controls; ^#^p < 0.05 vs cancer no cachexia.

Since autophagy is important also for the removal of dysfunctional organelles such as mitochondria^45^, we next examined the expression of Bnip3, Nix/Bnip3L, Parkin, and PINK1, proteins involved in the regulation of mitophagy^45^,^46^. Results obtained did not show statistically significant changes for both Bnip3 mRNA and protein levels, although a small trend toward increased Bnip3 protein levels was visible in cachectic cancer patients (Fig. 3A,C). Similarly, we did not observe any significantly different change in Nix/Bnip3L (both m-RNA and protein levels), although a trend to increase was observed in non-cachectic patients with respect to both controls and cachectic patients (mRNA) and in cancer patients (non-cachectic or cachectic) with respect to controls (protein) (Fig. 3B,D). Parkin m-RNA was significantly increased in non-cachectic patients with respect to controls. In cachectic patients, Parkin m-RNA levels were not different with respect to controls, but significantly lower than in non-cachectic patients (Fig. 4A). Parkin protein levels were reduced with respect to controls in non-cachectic patients (Fig. 4C). In cachectic patients, Parkin protein levels were significantly higher than in non-cachectic patients, but were not different from controls (Fig. 4C). With regard to PINK1, m-RNA levels were significantly reduced in cachectic cancer patients with respect to controls and non-cachectic patients, whereas protein levels were not significantly modulated, although a trend toward increase in non-cachectic patients was observed (Fig. 4B,D).

**Figure 5:**
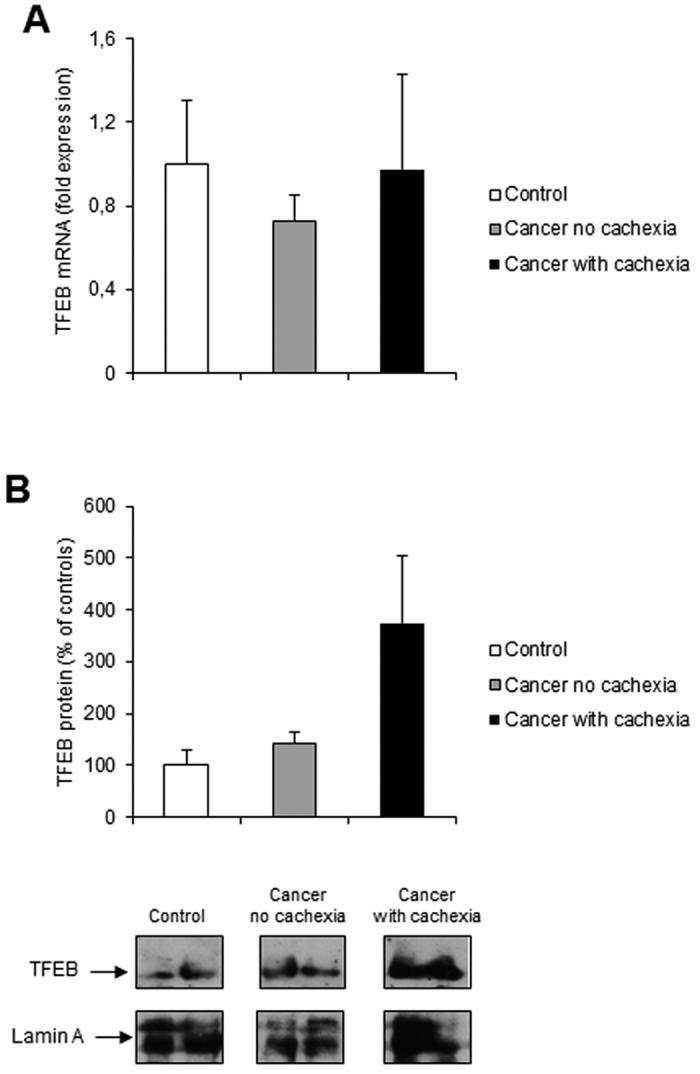
TFEB expression in rectus abdominis muscle of cancer and control patients. (**A**) TFEB mRNA levels were evaluated by real-time PCR (control, n = 11; non cachectic cancer patients, n = 16; cachectic cancer patients, n = 12); (**B**) TFEB nuclear protein levels were evaluated by western blotting (control, n = 4; non cachectic cancer patients, n = 5; cachectic cancer patients, n = 3): representative western blots for TFEB and Lamin A (loading control) are shown on the lower panel and densitometric quantifications of TFEB protein levels normalized to Lamin A are shown on the upper panel. Data (mean ± SEM) are expressed as percentage of controls.

Finally, since lysosomal dysfunction may contribute to defective autophagosome clearance and lead to their progressive accumulation, we assessed the expression of TFEB, a master upstream regulator of lysosomal biogenesis and autophagy, the activity of which is regulated by nutrient availability^47^. We found unchanged TFEB mRNA levels (Fig. 5A), and a trend toward higher TFEB nuclear protein levels in the skeletal muscle of cachectic cancer patients with respect to controls, although the differences did not reach statistical significance (Fig. 5B).

## Discussion

The present study aimed at clarifying whether autophagy is modulated in the skeletal muscle of cancer patients. Some data supporting such hypothesis are already available in the literature. Indeed, other studies have shown increased protein levels for Beclin-1 and ATG5, another protein involved in the early stages of autophagosome formation^48^,^49^, in the rectus abdominis of weight losing upper gastrointestinal or pancreatic cancer patients^33^ and a LC3B-II/LC3B-I ratio higher than in controls has been observed in the vastus lateralis of esophageal cancer patients^31^. Moreover, up-regulation of LC3B-I and LC3B-II protein expression has been shown also in the vastus lateralis muscle of cachectic lung cancer patients^32^, further supporting the concept that autophagy is induced in the skeletal muscle of patients affected by different cancer types.

The results shown in the present study confirm and further extend those of previous reports, showing that beclin-1 and LC3 B-II protein levels are increased in the skeletal muscle of cancer patients. As for beclin-1, its over-expression is not paralleled by increased transcript levels, suggesting that it might simply reflect an impaired protein removal. Since we cannot evaluate autophagic flux in cancer patients, this alternative hypothesis cannot be discarded. However, a comparable mismatch between m-RNA and protein levels was observed in experimental conditions where the autophagic flux was dynamically assessed^21^. The observation that also p62 protein levels are increased in the muscle of cancer patients suggests that an accumulation of unprocessed autophagosomes might occur, possibly due to exhaustion of the lysosomal degradative capacity. Importantly, such autophagosome accumulation could lead to dysfunction of cellular trafficking and marked abnormality of cytoskeleton organization in skeletal muscle fibers which may contribute to myofiber atrophy, loss of integrity and function^5^,^17^.

Accumulation of p62, despite autophagy induction, has been observed also in the gastrocnemius of mice bearing the C26 or Lewis Lung carcinoma and in rats implanted with the Yoshida AH-130 hepatoma^21^. Moreover, in the muscle of C26-bearing mice on day 14 after tumor implantation, the increased p62 protein levels were associated with reduced lysosomal cathepsin activities, suggesting that p62 accumulation may reflect disturbances in lysosomal function^21^. Conversely, in muscle biopsies of esophageal cancer patients increased LC3B-II/LC3B-I ratio and unchanged p62 protein level paralleled with increased cathepsin B and L activity^31^.

Mitophagy is a selective form of autophagy which plays an important role in removal of damaged mitochondria, and regulation of mitochondria turnover and abundance, according to changes in metabolic requirements^45^. Impairment of mitophagy is deleterious to muscle homeostasis and leads to the accumulation of damaged and dysfunctional mitochondria^15^,^46^. Although several markers of mitophagy have been assessed, the results obtained in the present study do not clearly define if this process is activated above physiological levels in the patient population analyzed. We did not observe significant changes in the expression of Bnip3 and Nix/Bnip3L, two molecules implicated in recruiting the autophagosomes to damaged mitochondria^4^,^46^. Since both markers show a trend toward increase, it is possible that sample size is not large enough to provide significant results. Unlike in our study, increased Bnip3 mRNA levels have been found in muscle biopsies of lung^32^ and upper gastrointestinal^50^ cancer patients. In both studies^32^,^50^, however, Bnip3 increase was detected as mRNA expression only, and whether such increase is reflected at the protein level is not known. In addition to the Bnip3-Nix/Bnip3L system, the Parkin/PINK1 pathway is also involved in inducing mitophagy^45^,^46^. The increase in Parkin transcript observed in non- cachectic cancer patients, paralleled by a reduction in protein levels, would argue in favor of an increased mitophagic flux. Indeed, Parkin is anchored to the mitochondrial membrane and is degraded together with mitochondria^51^. Conversely, Parkin m-RNA and protein levels in cachectic patients showed different trends, suggesting possibly impaired mitophagy activation in the cachectic muscle with respect to cancer patients without cachexia. The reduction of PINK1 expression in cachectic patients is consistent with findings in other forms of muscle depletion^52^ and further supports the view that mitophagy is impaired in human cancer cachexia. To our knowledge, this is the first description of a rather comprehensive panel of mitophagy markers in cancer patients. We acknowledge, however, that patient sample size is limited and does not allow to draw definite conclusion on whether mitophagy represents a significant contributor to cancer-related muscle wasting.

The transcription factor TFEB is a master regulator of autophagosome and lysosome biogenesis^47^. In normal conditions, TFEB is phosphorylated by different kinases, including ERK2 and mTORC1^47^,^53^, and is localized in the cytoplasm^47^,^53^,^54^. Upon starvation or lysosomal dysfunction, TFEB is dephosphorylated and rapidly translocates to the nucleus where it becomes active and regulates the expression of target genes^55^. To the best of our knowledge, this is the first report of TFEB expression in the skeletal muscle of cancer patients. In the present study, however, not enough evidence was observed to claim a difference in TFEB m-RNA and nuclear protein level, a finding which is likely the consequence of the high inter-individual variability.

In this investigation, we provide new insights into the role of autophagy in cancer cachexia. However, the results obtained should be interpreted with caution since the study has several limitations. First, we evaluated a small sample of cancer patients who are not necessarily representative of the entire population. Second, we could just offer an approximate estimate of the autophagic flux, but not an actual evaluation of the autophagic rate, as it is possible in experimental models^5^,^34^. However, the dynamic assessment of muscle autophagic flux performed in C26-bearing mice, yelded a pattern of autophagic markers similar to the one reported here, supporting the conclusions of the present study. Third, it is not possible to ascertain whether the cross-sectional determination of muscle autophagy performed in our cancer patients is really representative of the entire time course of the disease. In fact, variations in autophagy could occur in the same patient because of changes in physical activity, nutrient availability or other variables which could potentially affect the clinical outcome. In this regard, unfortunately, we did not record nutritional intake neither energy expenditure for patients enrolled in the present study and it is not known to which extent these factors may have contributed to the results obtained. We screened patients for the presence of anorexia by using specific questionnaires, but, because the percentage of patients complaining anorexia-related symptoms was quite similar among cachectic and non-cachectic cancer patients, it was not possible to draw any conclusions. Moreover, considering that most of the patients studied were aged >65 years, we cannot exclude that the impairment in autophagosome removal was at least partially secondary to age-related sarcopenia^4^,^21^,^22^.

Finally, we have also to acknowledge that body composition in the present study was assessed by BIA a technique that, in spite of its several advantages (portability, ease of use, non invasivity, low cost) may have some limitations. Among these, it must be considered that BIA largely relies on the use of regression equations, which makes of BIA an indirect method to assess body composition. In addition, assessment may be less precise in patients with fluid imbalance, such as those with edema, liver cirrhosis and chronic kidney disease^56^,^57^. It must be noted, however, that in the present study, BIA measurements did not affect the final interpretation of our findings, since they were purely descriptive and they have not been used for interpretation of data at the molecular level.

In conclusion, the results obtained in the present study suggest that autophagy is induced in the skeletal muscle of cachectic cancer patients although autophagosome clearance appears to be impaired, possibly because of exhaustion of the lysosomal degradative capacity. Mitophagy also seems impaired, although the results obtained are not conclusive.

The real challenge for future studies will be to better clarify if and to what extent is autophagy a determinant of cancer-related muscle wasting and whether targeting this pathway^58^,^59^ could represent a therapeutic strategy for cancer cachexia. These questions will remain unanswered, until the “yin and yang” of this obscure yet fascinating mechanism will be fully elucidated.

## Additional Information

**How to cite this article**: Aversa, Z. *et al*. Autophagy is induced in the skeletal muscle of cachectic cancer patients. *Sci. Rep*. **6**, 30340; doi: 10.1038/srep30340 (2016).

## Supplementary Material

Supplementary Information

## Figures and Tables

**Table 1:** Characteristics of the study population.

Variables	Cancer patients	Control patients
Subjects (*n*)	29	11
Age (*years*)	68 ± 2	63 ± 4
Sex		
Male (*n*)	17	7
Female (*n*)	12	4
Cancer site		—
Colon-rectum (*n*)	14	
Pancreas (*n*)	5	
Stomach (*n*)	4	
Miscellaneous (*n*)	6	
Cancer stage		
I-II (*n*)	9	
III-IV (*n*)	20	
Height (*m*)	1.64 ± 0.02	1.67 ± 0.03
Weight at diagnosis (*kg*)	71.3 ± 2.9	76.7 ± 4.7
BMI at diagnosis (*kg/m*^*2*^)	26.6 ± 0.94	27.4 ± 1.5
% body weight loss over last 6 months	6.7 ± 1.5	—
Patients with anorexia (*n*)	11	—
Cachexia classification		
Pre-cachexia (*n*)	3	—
Cachexia (*n*)	12	—
No pre-cachexia/no cachexia (*n*)	14	—

**Table 2:** Anthropometric and BIA-derived parameters in patients stratified for cachexia.

Variables	Control patients	Non cachectic cancer patients	Cachectic cancer patients
BMI (*kg/m*^*2*^)	27.4 ± 1.5	28.3 ± 1.1	24.5 ± 1.5^a^,^b^
% body weight loss over last 6 months	—	0.6 ± 0.3	13.3 ± 2.00^b^
FFM (*kg*)	50.96 ± 3.00	50.79 ± 2.24	43.13 ± 2.38^a^,^b^
FFMI (*kg/m*^*2*^)	18.08 ± 0.71	18.56 ± 0.49	16.38 ± 0.74^b^
FM (*kg*)	25.73 ± 2.89	26.33 ± 2.57	24.89 ± 4.80
FMI (*kg/m*^*2*^)	9.28 ± 1.10	9.69 ± 0.91	9.24 ± 1.55
Phase angle (°)	5.63 ± 0.29	5.75 ± 0.37	5.09 ± 0.39

^a^p < 0.05 vs control patients.

^b^p < 0.05 vs non cachectic cancer patients. Abbreviations: BIA, bioelectrical impedance analysis.

BMI, Body Mass Index; FFM, Fat Free Mass; FFMI, Fat Free Mass Index; FM, Fat Mass; FMI, Fat Mass Index.

**Table 3:** Biochemical and hematological indices in patients stratified for cachexia.

Variables	Control patients	Non cachectic cancer patients	Cachectic cancer patients
Serum total protein (*g/dL*)	6.8 ± 0.2	6.7 ± 0.3	6.3 ± 0.2
Serum albumin (*g/dL*)	4.2 ± 0.1	3.8 ± 0.2	3.5 ± 0.2^a^
C-reactive protein (*mg/dL*)	0.45 ± 0.17	1.92 ± 1.04	2.07 ± 0.98
Ferritin (*ng/mL*)	125 ± 32	198 ± 61	156 ± 31
Serum iron (*μg/mL*)	109 ± 16	63 ± 8	70 ± 10
Serum creatinine (*mg/dL*)	0.95 ± 0.06	0.96 ± 0.08	0.8 ± 0.05
Total cholesterol (*mg/dL*)	179 ± 21	168 ± 11	178 ± 11
HDL cholesterol (*mg/dL*)	51 ± 2	45 ± 6	44 ± 5
LDL cholesterol (*mg/dL*)	125 ± 18	92 ± 10	114 ± 12
Triglycerides (mg/dL)	100 ± 15	123 ± 15	109 ± 11
Haemoglobin (*g/dL*)	14.4 ± 0.4	12.4 ± 0.5^a^	12.1 ± 0.4^a^
White cell count (*x10*^*3*^*/cm*^3^)	6.17 ± 0.3	7.7 ± 0.99	5.6 ± 0.5
Total lymphocytes count (*x10*^*3*^*/cm*^3^)	1.9 ± 0.096	1.7 ± 0.19	1.4 ± 0.20^a^

^a^p < 0.05 vs control patients.
