# Supplementary material for: Autophagy is induced in the skeletal muscle of cachectic cancer patients
Source: Sci Rep. 2016 Jul 27;6:30340. doi: 10.1038/srep30340 (PMC4962093; doi:10.1038/srep30340)
Supplement: Supplementary Information [file srep30340-s1.pdf]

Supplementary Information

AUTOPHAGY IS INDUCED IN THE SKELETAL MUSCLE OF CACHECTIC CANCER PATIENTS

Zaira Aversa[1*], Fabrizio Pin[2,3*], Simone Lucia[1], Fabio Penna[2,3], Roberto Verzaro[4], Maurizio Fazi[4], Giuseppina Colasante[5], Andrea Tirone[5], Filippo Rossi Fanelli[1], Cesarina Ramaccini[1], Paola Costelli[2,3], Maurizio Muscaritoli[1]

[1]Department of Clinical Medicine, Sapienza University of Rome, Rome, Italy

[2]Department of Clinical and Biological Sciences, University of Turin, Turin, Italy

[3]Interuniversity Institute of Myology, Italy

[4] Department of Surgery, M.G. Vannini Hospital, Rome, Italy

[5]UOSA Chirurgia Bariatrica, Azienda Ospedaliera Universitaria Senese, Siena, Italy

*Zaira Aversa and Fabrizio Pin equally contributed.

Corresponding author:

Maurizio Muscaritoli, Department of Clinical Medicine, Sapienza, University of Rome, Viale dell'Università 37, 00185 Rome, Italy (e-mail: maurizio.muscaritoli@uniroma1.it) ; or

Paola Costelli, Department of Clinical and Biological Sciences, University of Turin - Corso Raffaello 30, 10125 Turin, Italy (e-mail: paola.costelli@unito.it).

Supplementary Table S1. Oligonucleotide sequences used for real-time PCR

Gene	*NCBI Reference Sequence*	*Primer sequence*
**Beclin-1**	NM_ 003766.3	FW: AGGAACTCACAGCTCCATTAC<br>RW: AATGGCTCCTCTCCTGAGTT
**p62/SQSTM1**	NM_003900.4	FW: ATCGGAGGATCCGAGTGT<br>RW: TGGCTGTGAGCTGCTCTT
**LC3B**	NM_022818.4	FW: GCCGCACCTTCGAACAAA<br>RW: TCGTTCTATTATCACCGGGATTTT
**Bnip3**	NM_ 004052.2	FW: CTGGACGGAGTAGCTCCAAG<br>RW: GCCCTGTTGGTATCTTGTGG
**Nix/Bnip3L**	NM_004331.2	FW: GATGTGGAAATGCACACCAG<br>RW: GACCAGTCTGATACCCAGTCC
**PINK1**	NM_032409.2	FW: TGGTCGACTACCCTGATGTG<br>RW: CGCAGGGTACAGGGATAGTT
**Parkin**	NM_004562.2	FW: TCGCAACAAATAGTCGGAAC<br>RW: GAACAAACTGCCGATCATTG
**TFEB**	NM_007162.2	FW: GGTGCAGTCCTACCTGGAGA<br>RW: GTGGGCAGCAAACTTGTTCC
**GAPDH**	NM_002046.3	FW: CCACTCCTCCACCTTTGAC<br>RW: ACCCTGTTGCTGTAGCCA
**TBP**	NM_003194	FW: TGCACAGGAGCCAAGAGTGAA<br>RW: CACATCACAGCTCCCCACCA

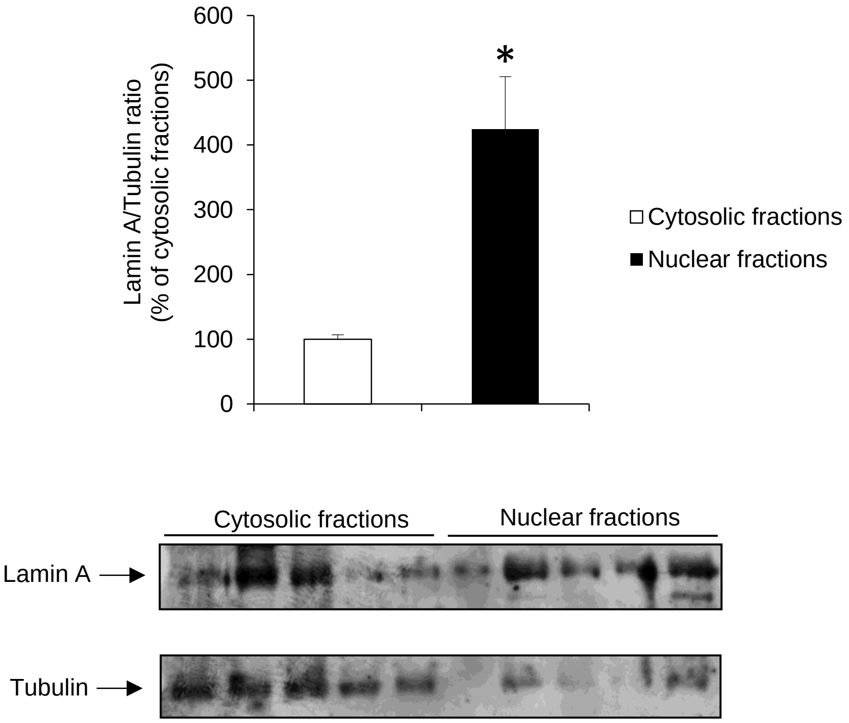

Supplementary Figure S1. Purity of the cytosolic and nuclear fractions. (A) Lamin A/Tubulin ratio was evaluated by western blotting in cytosolic (n=5) and nuclear (n=5) fractions: representative western blots for Lamin A and Tubulin are shown on the lower panel and densitometric quantifications of Lamin A/Tubulin ratio are shown on the upper panel. Data (mean ± SEM) are expressed as percentage of cytosolic fractions. Significance of the differences: *p<0.05 vs cytosolic fractions.

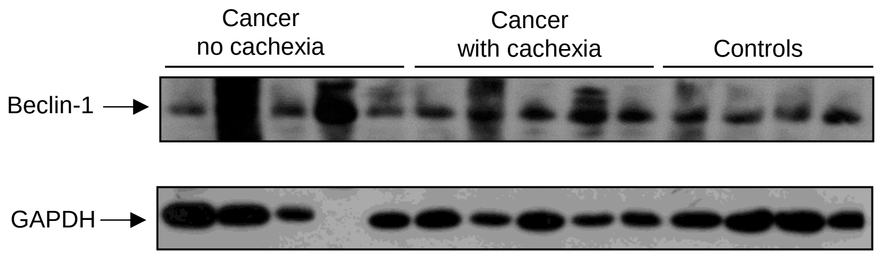

Supplementary Figure S2. Beclin-1 protein expression in rectus abdominis muscle of cancer and control patients. Whole blot including the representative patterns selected for beclin-1 expression in Fig. 1C (lanes 1 and 3).
